# Assessment of Public Awareness and Prevalence of Asthma Comorbidities in Al-Qunfudhah, Saudi Arabia

**DOI:** 10.7759/cureus.85389

**Published:** 2025-06-05

**Authors:** Abdulrahman Saleh Alrashide, Mohammed Qati Alessa, Mohamed Elhefny, Mahmoud Abutaleb Alsayed, Sami Yaseen Alamri, Ahmed Bellgasim Alfageh, Ahmed Balghith alzubaidi

**Affiliations:** 1 Medical College, Umm Al-Qura University, Makkah, SAU; 2 Cancer and Molecular Biology, National Cancer Institute, Cairo University, Cairo, EGY; 3 Genetics, Umm Al-Qura University, Makkah, SAU; 4 Medical College, Umm Al-Qura University, Al-Qunfudhah, SAU; 5 Medicine and Surgery, Umm Al-Qura University, Al-Qunfudhah, SAU; 6 Medical Collage, Umm Al-Qura University, Makkah, SAU

**Keywords:** allergic rhinitis, al-qunfudhah, asthma prevalence, comorbidities, public health, s: asthma awareness, saudi arabia

## Abstract

Background

Asthma is a globally prevalent chronic respiratory disease with rising incidence, particularly in developing countries, where underdiagnosis and poor disease control are common. In Saudi Arabia, regional disparities in asthma awareness and healthcare accessibility contribute to inconsistent disease outcomes. Al-Qunfudhah, a semi-urban coastal city in western Saudi Arabia, lacks localized data on asthma prevalence and associated comorbidities, despite its unique environmental challenges such as maritime humidity and seasonal dust storms.

Objectives

This study aimed to assess the level of public awareness about asthma, identify the prevalence of self-reported asthma and associated comorbidities, and evaluate demographic correlations with asthma-related knowledge among residents of Al-Qunfudhah.

Methods

A descriptive cross-sectional study was conducted between January and March 2025, involving 540 adult residents of Al-Qunfudhah recruited via convenience sampling through an online Google form. A prevalidated, structured Arabic and English questionnaire assessed sociodemographic data, asthma knowledge and awareness, comorbid conditions, and management practices. Statistical analyses were performed using SPSS version 26 (IBM Corp., Armonk, USA), with chi-square tests to examine associations (p < 0.05 considered significant).

Results

Most participants were male (64.6%), aged 46-60 years (28.9%), and held a university degree or higher (74.3%). Although 94.4% correctly identified asthma as a lung disease, only 66.9% could recognize all common symptoms, with fewer recognizing persistent cough (21.1%) and dyspnea (43.0%). Approximately 20.2% had a prior asthma diagnosis, and among them, 69.8% had been diagnosed for over five years. Notably, 74.3% were aware of preventive measures, yet only 14.6% reported regular use of asthma medications, and 50.2% had not received inhaler technique instruction. Comorbidities were prevalent: allergic rhinitis (20.2%), obesity (5.6%), and gastroesophageal reflux disease (GERD) (3.7%) were the most reported. Awareness was significantly higher among those previously diagnosed with asthma, individuals aged 46-60 years, and those with intermediate education.

Conclusion

While general awareness of asthma in Al-Qunfudhah is relatively high, significant gaps remain in symptom literacy, medication use, and understanding of asthma-related comorbidities. These findings highlight an urgent need for community-based asthma education programs tailored to regional demographics. Targeted public health initiatives that promote accurate symptom recognition, proper inhaler usage, and integrated management of comorbidities are essential to improving asthma outcomes in underserved areas like Al-Qunfudhah.

## Introduction

Asthma is a chronic inflammatory disease of the lower airways, characterized by recurrent episodes of wheezing, dyspnea, chest tightness, and coughing, which are typically variable and reversible either spontaneously or with appropriate treatment [1]. It is a condition that imposes a substantial burden on patients, families, and healthcare systems worldwide. According to the Global Initiative for Asthma (GINA, 2024), an estimated 300 million individuals currently live with asthma, with projections indicating this number could exceed 400 million by 2025 due to ongoing urbanization, increasing exposure to environmental pollutants, and widespread adoption of sedentary lifestyles [1].

Despite extensive global awareness of asthma, the disease remains widely underdiagnosed and inadequately controlled, particularly in low- and middle-income countries (LMICs). Multiple factors contribute to this, including insufficient public health infrastructure, limited accessibility to specialized care, socioeconomic disparities, and suboptimal disease literacy among the general population [2]. Effective asthma management is not solely reliant on clinical diagnosis and pharmacotherapy but also heavily depends on the public’s understanding of the disease. Comprehensive awareness of asthma symptoms, triggers, management strategies, and associated comorbidities is essential for early detection, prevention of exacerbations, and reducing asthma-related morbidity and mortality [3].

In the Kingdom of Saudi Arabia (KSA), asthma constitutes a significant public health concern. Reported prevalence rates vary between 8% and 25%, with the highest figures noted among children and adolescents [4, 5]. Contributing factors include rapid urbanization, frequent exposure to dust storms, high levels of airborne pollutants, and lifestyle habits such as tobacco smoking [6, 7, 8]. Furthermore, KSA experiences marked regional disparities in healthcare accessibility and environmental exposures, which influence asthma prevalence and disease burden [9, 10, 11]. However, even with this substantial burden, there remains a paucity of localized data on asthma prevalence, awareness, and management practices, particularly in smaller cities and rural communities [12].

Al-Qunfudhah, a coastal city located in the western region of Saudi Arabia, represents one such underexplored locality. The city's semi-rural structure, compounded by its unique environmental context, including maritime humidity, seasonal dust storms, and occasional air stagnation, creates conditions potentially conducive to respiratory illnesses, including asthma. However, epidemiological research in Al-Qunfudhah has been limited, with little known about the population’s level of asthma awareness or the local prevalence of asthma-associated comorbidities [13, 14].

Asthma is not an isolated disease; it frequently coexists with multiple chronic conditions that complicate diagnosis, impact disease control, and exacerbate symptoms. Common comorbidities include allergic rhinitis, obesity, gastroesophageal reflux disease (GERD), eczema, anxiety disorders, and cardiovascular diseases. Allergic rhinitis is present in up to 80% of asthma patients and has a bidirectional relationship with asthma - each condition can exacerbate the other [14, 15]. Obesity is associated with increased asthma severity, reduced responsiveness to inhaled corticosteroids, and higher healthcare utilization rates [16, 17]. GERD, though often underrecognized, can induce asthma-like symptoms and worsen bronchial hyperreactivity due to micro-aspiration and vagal reflexes [18, 19]. These interactions underscore the need for integrated management approaches that address both asthma and its comorbidities concurrently.

Importantly, the general public’s awareness of such comorbid conditions and their potential impact on asthma control is often limited. A lack of recognition and underreporting of comorbidities may hinder optimal disease management and lead to recurrent exacerbations or inappropriate treatment regimens. Educating the public about the interconnected nature of asthma and its comorbidities is therefore essential for improving outcomes, reducing healthcare costs, and promoting quality of life among individuals with asthma [20], [21], [22].

Given these considerations, the present study was designed to assess public awareness of asthma and the prevalence of related comorbidities in Al-Qunfudhah. Specifically, the study aimed to (1) evaluate general knowledge about asthma and its symptoms, (2) explore the sources and quality of asthma-related information accessible to the community, (3) determine the self-reported prevalence of asthma and comorbid conditions, and (4) examine the relationship between demographic characteristics and asthma awareness levels. The rationale for conducting this research in Al-Qunfudhah stems from the urgent need to generate localized data that can inform health policy, guide community outreach efforts, and support the design of culturally and contextually appropriate asthma education programs [23].

The significance of this research lies in its potential to bridge critical gaps in asthma surveillance and health education in Saudi Arabia. While major urban centers like Riyadh and Jeddah have been the focus of asthma-related investigations [12], there is a dearth of comparable data from semi-urban or rural areas, which are often underserved by the healthcare system. Public health efforts cannot be one-size-fits-all; understanding region-specific factors is essential for the creation of effective, equitable interventions. For instance, integrating asthma awareness with primary care visits, school health programs, and mosque-based education initiatives may be more effective in communities like Al-Qunfudhah than in large metropolitan areas [24, 25].

Furthermore, the recent acceleration in digital health tools, including telemedicine, health apps, and remote monitoring, provides an unprecedented opportunity to enhance asthma education and management in geographically dispersed regions. Leveraging these technologies, alongside traditional community-based outreach, can promote wider dissemination of health information, particularly in contexts where healthcare access remains a challenge [26, 27].

## Materials and methods

Study design and setting

This study employed a descriptive, cross-sectional design to investigate public awareness of asthma and the prevalence of self-reported asthma-related comorbidities within the general population of Al-Qunfudhah, Saudi Arabia. Data collection occurred between January and March 2025.

Study population and sampling

Inclusion criteria: The individuals included in this study were required to be currently residing in Al-Qunfudhah and be 18 years of age or older.

Exclusion criteria: Individuals were excluded from the study if they did not reside in Al-Qunfudhah or were younger than 18 years of age.

A total of 540 participants were recruited using a convenience sampling method via an online Google form.

Data collection instrument

A pretested and validated structured questionnaire, developed in Arabic and English, served as the data collection instrument. The questionnaire encompassed four key sections:

Sociodemographic Data: This section collected information on participants' age, gender, and education level.

Asthma Knowledge and Awareness: This section assessed participants' understanding of basic asthma concepts, their ability to identify common symptoms, and their knowledge of potential triggering factors.

Management and Prevention Practices: This section explored participants' reported use of asthma medications, their adoption of preventive strategies, and the sources from which they obtained asthma-related information.

Comorbidities and Lifestyle Factors: This section gathered data on the self-reported presence of several conditions potentially associated with asthma, including allergic rhinitis, gastroesophageal reflux disease (GERD), obesity, eczema, heart disease, diabetes, and hypertension, as well as participants' smoking status.

Prior to data collection, the questionnaire underwent review by local healthcare experts to ensure content validity and cultural appropriateness. A pilot study involving 20 participants was conducted to assess the clarity, comprehensibility, and reliability of the instrument. Feedback from the pilot study was incorporated to refine the final version of the questionnaire.

Ethical considerations

Ethical approval was granted from the Umm Al-Qura University ethics committee (approval number: HAPO-02-K-012-2025-01-2507). Participation in the study was entirely voluntary, and informed consent was obtained electronically from all participants prior to their inclusion. Anonymity was maintained throughout the data collection process, and all collected data were used solely for the purposes of this research.

Statistical analysis

All collected responses were coded and analyzed using IBM SPSS Statistics software, version 26 (IBM Corp., Armonk, NY, USA). Descriptive statistics, including frequencies and percentages, were used to summarize categorical variables. To examine the relationships between demographic variables and levels of asthma awareness, the Chi-square (χ2) test was employed. A p-value of less than 0.05 (p<0.05) was considered to indicate statistical significance.

## Results

Demographic information

This study included 540 participants after excluding five who didn't consent to participate. The demographic characteristics of the study participants revealed that the majority (28.9%) were aged between 46 and 60 years. Most participants were males (64.6%), and the predominant educational level among them was a university degree or higher (74.3%) (Table 1).

**Table 1:** Demographic data

Parameter	Category	N	%
Age (years)	18-25	158	29.26%
	26-35	92	17.04%
	36-45	111	20.56%
	46-60	156	28.89%
	> 60	23	4.26%
Gender	Female	191	35.4%
	Male	349	64.6%
Educational level	No formal education	5	0.9%
	Primary education	12	2.2%
	Intermediate education	10	1.9%
	Secondary education	112	20.7%
	University degree or higher	401	74.3%

Knowledge about asthma

**Figure 1:**
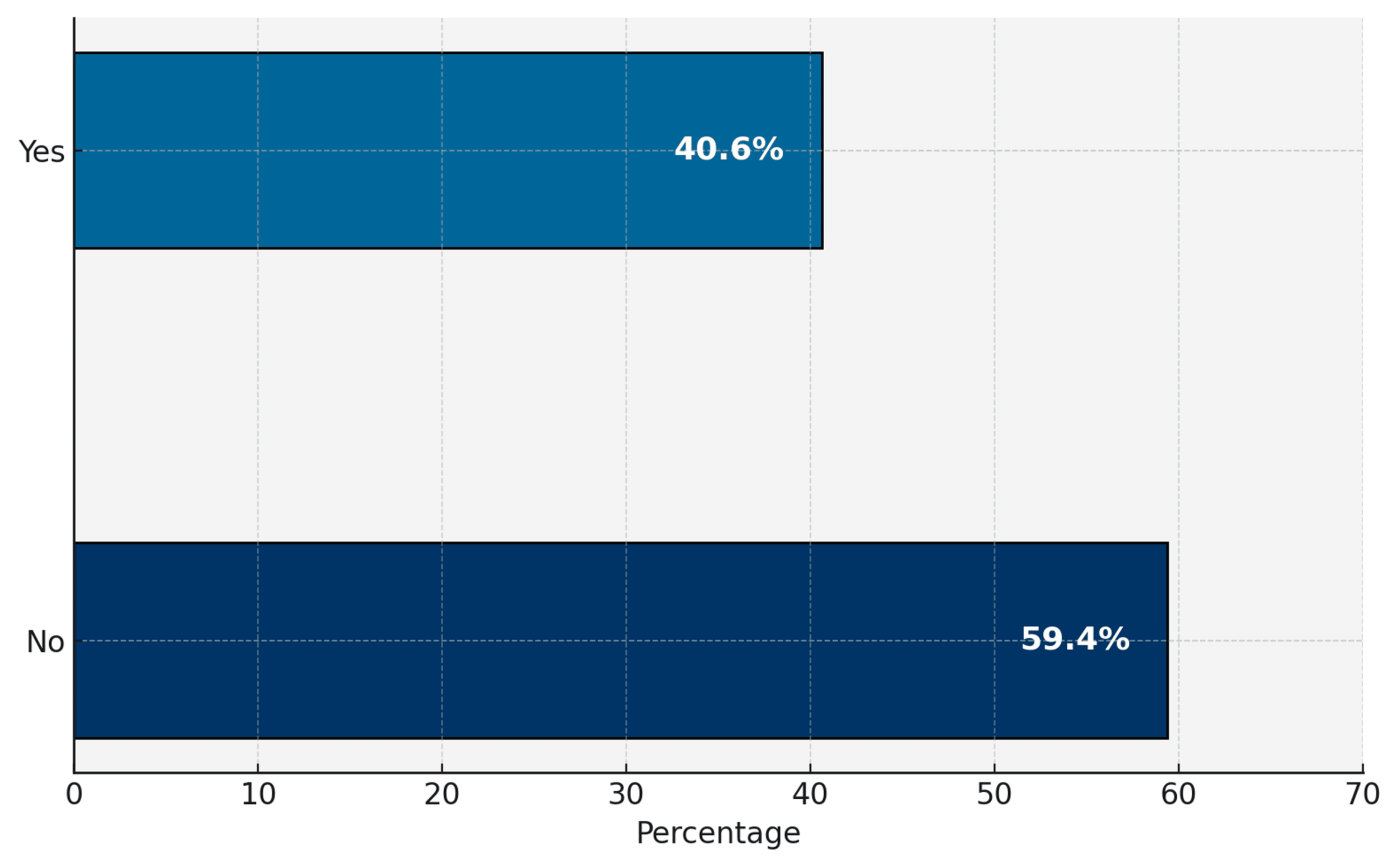
Bar chart showing the regularity of exercising

**Figure 2:**
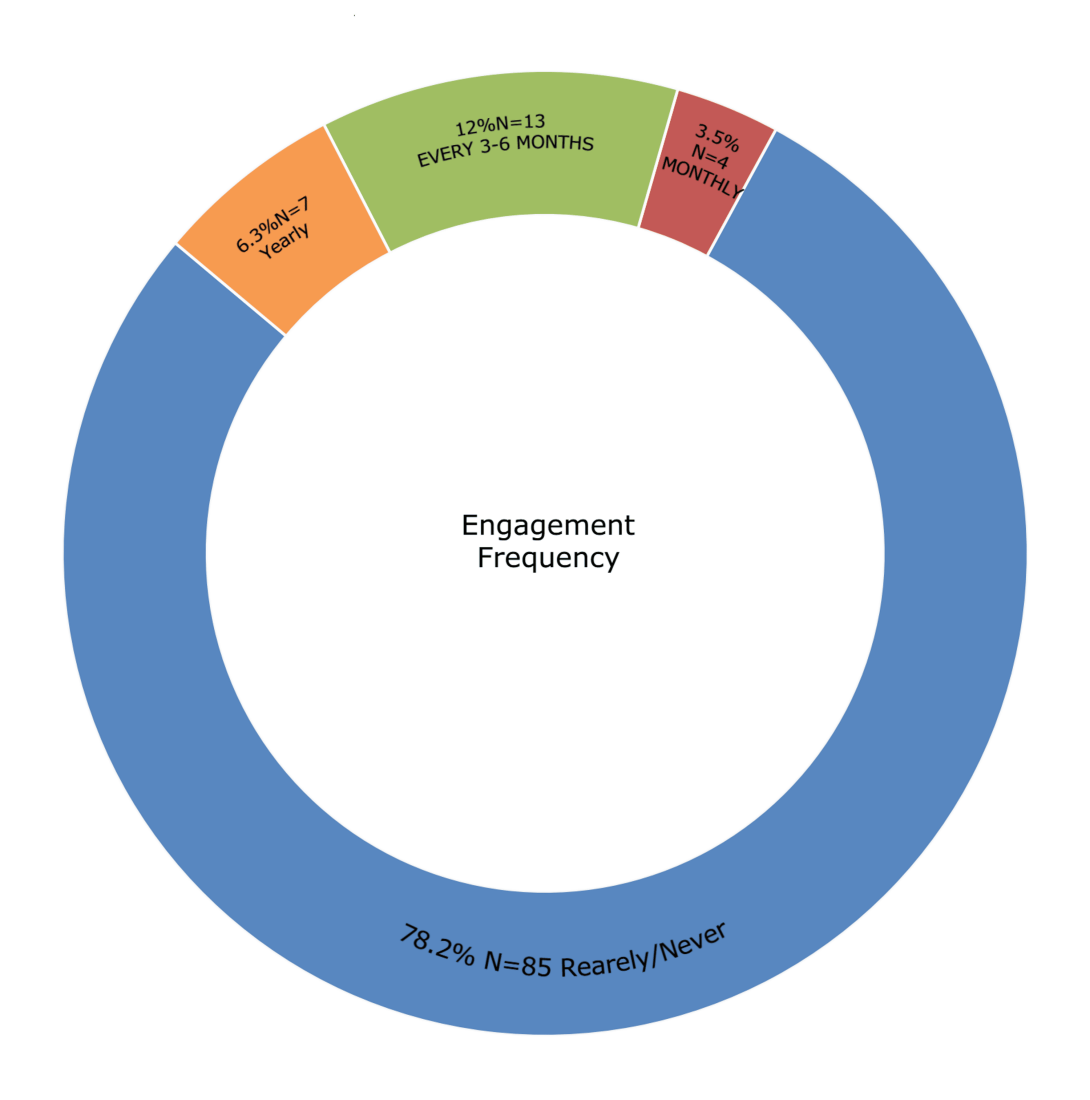
Pie chart showing the frequency of visiting asthma doctor

The majority of participants (94.4%) correctly identified asthma as a condition affecting the lungs and causing difficulty breathing due to airway obstruction. Most respondents (79.8%) reported never having been diagnosed with asthma by a specialist. Among those diagnosed, 69.8% stated they were diagnosed more than five years ago. Regarding asthma symptoms, 66.9% of participants recognized all common symptoms, and 43.0% of them recognized difficulty breathing, while 42.8% rated their knowledge of asthma symptoms as good. Most participants (79.3%) reported not currently experiencing asthma symptoms. Air pollution and smoke were identified as the most common aggravating factors (35.4%). The majority (72.0%) indicated that they neither smoked nor lived with a smoker, and 49.6% reported that no one in their family had asthma (Table 2). Regarding the regularity of exercising, 59.4% of participants did not exercise regularly (Figure 1). 78.1% of participants rarely or never visited an asthma doctor (Figure 2). 

**Table 2:** Knowledge about asthma

Parameter	Category	N	%
What is asthma?	A condition affecting the lungs and causing difficulty breathing due to airway obstruction	510	94.4%
	A condition affecting the nervous system and causing loss of balance	17	3.1%
	A disease causing inflammation of blood vessels and affecting the heart	11	2.0%
	A disease affecting the joints and causing severe body pain	2	0.4%
Have you ever been diagnosed with asthma by a specialist?	No	431	79.8%
	Yes	109	20.2%
If yes, when were you diagnosed?	Less than a year ago	17	16.0%
	From 1-5 years ago	15	14.2%
	More than 5 years ago	74	69.8%
What are the most common symptoms of asthma you are aware of? (Select all that apply)	Chest tightness	154	28.5%
	Difficulty breathing	232	43.0%
	Wheezing	137	25.4%
	Persistent coughing	114	21.1%
	Sleep apnea	92	17.0%
	All of the above	361	66.9%
How would you rate your knowledge of asthma symptoms?	Poor	41	7.6%
	Moderate	152	28.1%
	Good	231	42.8%
	Excellent	116	21.5%
Are you currently experiencing asthma symptoms?	No	428	79.3%
	Yes, occasionally	88	16.3%
	Yes, frequently	24	4.4%
What factors worsen your asthma symptoms? (Select all that apply)	Dust and pollen	157	29.1%
	Strong odors (e.g., perfumes)	122	22.6%
	Pets (e.g., cats, dogs)	103	19.1%
	Air pollution and smoke	191	35.4%
	Colds and flu	128	23.7%
	All of the above	407	75.4%
Do you smoke or live with someone who smokes?	No, I don’t smoke or live with a smoker	389	72.0%
	No, I don’t smoke but live with a smoker	100	18.5%
	Yes, I smoke	51	9.4%
Does anyone in your family have asthma?	No	268	49.6%
	Yes	202	37.4%
	I don't know	70	13.0%

Awareness about asthma

The majority of participants (34.3%) received information about asthma from a doctor, while most (50.2%) did not need instructions on how to use an inhaler. The vast majority (85.4%) did not use asthma medications, and 30.0% rated the availability of asthma medications as excellent. Regarding asthma prevention, 74.3% of respondents were aware of all preventive measures, including avoiding triggers, using prescribed medications, maintaining a healthy weight, and quitting smoking. Nearly half (49.8%) reported that asthma did not affect their daily activities. Most participants (73.1%) reported no comorbidities, and among those who did, allergic rhinitis was the most common (20.2%). Additionally, 69.4% of respondents did not avoid activities or environments due to asthma. Among those who did, the most common reasons were pollution and smoke (28.4%) and hard exercises (24.2%) (Table 3).

**Table 3:** Awareness about asthma

Parameter	Category	N	%
Have you received information about asthma?	No	132	24.4%
	Yes, from a doctor	185	34.3%
	Yes, from workshops or awareness campaigns	53	9.8%
	Yes, from the internet	170	31.5%
Have you received instructions on how to use an inhaler for asthma?	No	31	28.5%
	Yes	23	21.3%
	I didn’t need it	55	50.2%
Do you use asthma medications?	No	461	85.4%
	Yes	79	14.6%
How would you rate the availability of asthma medications when needed?	I don’t use asthma medications	220	40.7%
	Poor	21	3.9%
	Good	137	25.4%
	Excellent	162	30.0%
What asthma prevention methods are you aware of? (Select all that apply)	Avoiding triggers like smoke and dust	189	35.0%
	Using prescribed medications	129	23.9%
	Maintaining a healthy weight	73	13.5%
	Quitting smoking	118	21.9%
	All of the above	401	74.3%
Does asthma affect your daily activities?	No	54	49.8%
	Sometimes	22	20.4%
	Often	22	20.4%
	Always	10	9.4%
Do you have any comorbidities alongside asthma? (Select all that apply)	None	395	73.1%
	Gastroesophageal reflux disease (GERD)	20	3.7%
	Obesity	30	5.6%
	Allergic rhinitis	109	20.2%
	Eczema	26	4.8%
	Heart disease	10	1.9%
	Diabetes	24	4.4%
	High blood pressure	32	5.9%
Do you avoid certain activities or environments due to asthma?	No	375	69.4%
	Yes	165	30.6%
If yes, please specify	Pollution and smoke	27	28.4%
	Hard exercises	23	24.2%
	Crowding	3	3.2%
	Cold	2	2.1%
	High places	1	1.1%
	Other	33	34.7%
	None	6	6.3%

Relation of demographic information and asthma diagnosis with awareness about asthma

The analysis showed that the highest percentage of asthma awareness was among participants aged 46-60 years (77.6%). Males had a slightly higher awareness of asthma (75.9%) compared to females (74.9%). Participants with intermediate education reported the highest asthma awareness (90.0%). Those diagnosed with asthma by a specialist had a significantly higher awareness (90.8%) compared to those not diagnosed (71.7%), with a statistically significant P-value (<0.001) (Table 4).

**Table 4:** Relation of demographic information and asthma diagnosis with awareness about asthma *P < 0.05 (significant) Chi-square value (used for comparison of awareness among different demographics)

Parameter	Category	No		Yes		P-value	Chi-square value
		N	%	N	%		
Age	18-25	42	23.4%	116	76.6%	0.869	1.857
	26-35	26	28.3%	66	71.7%		
	36-45	26	23.4%	85	76.6%		
	46-60	35	22.4%	121	77.6%		
	> 60 years	7	30.4%	16	69.6%		
Gender	Female	48	25.1%	143	74.9%	0.784	0.075
	Male	84	24.1%	265	75.9%		
Educational level	No formal education	1	20.0%	4	80.0%	0.723	2.071
	Primary education	3	25.0%	9	75.0%		
	Intermediate education	1	10.0%	9	90.0%		
	Secondary education	24	21.4%	88	78.6%		
	University degree or higher	103	25.7%	298	74.3%		
Have you ever been diagnosed with asthma by a specialist?	No	122	28.3%	309	71.7%	< 0.001	17.242
	Yes	10	9.2%	99	90.8%		

## Discussion

This study provides a comprehensive assessment of asthma awareness and the prevalence of related comorbidities among residents of Al-Qunfudhah, a region of Saudi Arabia that is underrepresented in national health data.

Our findings contribute to a growing body of evidence showing that while general awareness of asthma is relatively high - 94.4% of participants correctly identified asthma as a lung condition - critical gaps persist in symptom recognition, comorbidity awareness, treatment adherence, and proactive health-seeking behaviors.

Asthma awareness and the knowledge-behavior gap

Although general awareness of asthma was encouraging, only 66.9% of participants could correctly identify all common asthma symptoms. A notable 21.1% recognized persistent coughing and 43.0% identified difficulty breathing as symptoms - figures that underscore a gap between conceptual awareness and functional health literacy. This finding aligns with regional surveys in the Middle East and North Africa (MENA), where patients often misunderstand symptom profiles, leading to delayed care and poor disease management [14].

Effective asthma control depends not only on diagnosis and treatment but also on patient education and self-management. The Global Asthma Report (2024) emphasizes that patient education should be symptom-specific and culturally tailored, rather than solely focused on defining the disease [1]. Our findings suggest that educational interventions in Al-Qunfudhah must move beyond general awareness campaigns and instead focus on enhancing understanding of early warning signs and exacerbation triggers [2].

Comorbidities and their clinical relevance

Asthma frequently coexists with other chronic conditions such as allergic rhinitis (20.2% of our participants), obesity (5.6%), GERD (3.7%), eczema (4.8%), and hypertension (5.9%). These comorbidities contribute to asthma severity, increased healthcare utilization, and reduced quality of life. Allergic rhinitis, in particular, shares a pathophysiological basis with asthma - both involve IgE-mediated inflammation [13].

Furthermore, the relationship between obesity and asthma is well established. Obesity may reduce lung volume and exacerbate airway inflammation, and obese individuals often respond poorly to standard controller [15, 16]. GERD can mimic or worsen asthma symptoms by increasing bronchial reactivity, making it an important, yet often overlooked, contributor to poor asthma control [18].

The presence of these comorbidities - and the apparent lack of awareness among participants regarding their impact - reinforces the importance of holistic asthma management. Clinicians should routinely screen for these conditions, and health promotion strategies must incorporate information on the interconnected nature of chronic diseases [20, 21].

Medication use and inhaler technique

One of the most concerning findings was that only 14.6% of participants reported using asthma medications, and more than half (50.2%) claimed they did not require instruction on inhaler use. These data suggest a severe gap in patient education, especially considering the critical role that correct inhaler technique plays in effective drug delivery.

Research shows that improper inhaler use contributes significantly to poor asthma outcomes and is associated with increased emergency department visits [17, 19]. This gap may be attributed to a lack of access to asthma educators, inconsistent counseling during clinical encounters, or cultural misconceptions regarding pharmaceutical therapy.

Structured education programs, ideally integrated within primary care, are necessary to improve adherence and technique. These should include visual aids, hands-on demonstrations, and follow-up assessments to ensure long-term competence and confidence among patients [16, 21].

Sociodemographic correlates of awareness

Our results indicate that asthma awareness was highest among older adults (46-60 years) and those with previous asthma diagnoses. This finding likely reflects greater personal experience with the disease and increased interaction with healthcare services. However, younger individuals (especially under 25 years) demonstrated lower awareness, echoing patterns seen in other Saudi and Gulf populations where younger age correlates with reduced health literacy and lower engagement [28].

This suggests a clear target for intervention: educational strategies should be customized by age and literacy level, employing engaging, youth-friendly platforms like social media, mobile apps, and school-based programs. Empowering younger populations with preventive knowledge could reduce long-term disease burden [13, 29].

Health-seeking behaviors and use of technology

Another key finding was the underutilization of asthma-related healthcare services - 78.1% of participants rarely or never visited asthma specialists. Coupled with a relatively low rate of engagement in physical activity (59.4%), this reflects a pattern of reduced health-seeking behavior common in semi-urban Saudi communities [3].

In this context, digital health solutions, including mobile health (mHealth) apps and telemedicine, could bridge critical access gaps. These tools became especially prominent during the COVID-19 pandemic and remain underutilized in routine asthma care. Digital health interventions can significantly enhance monitoring, adherence, and patient education, particularly in resource-limited settings [30].

Comparisons with other regions in Saudi Arabia

When compared to similar studies conducted in Riyadh, Jeddah, and the Eastern Province, asthma awareness levels in Al-Qunfudhah appear lower, likely due to differences in healthcare access, urbanization, and the extent of public health outreach [29]. These disparities underscore the necessity of investing in targeted educational campaigns that reach underserved and geographically isolated populations [3, 5].

Limitations and future research

This study has several limitations. First, the cross-sectional design prevents the establishment of causality. Second, reliance on self-reported data may introduce recall bias, particularly regarding disease diagnosis and medication use. Third, online data collection may exclude individuals with limited digital access or literacy.

Future research should adopt longitudinal designs to assess changes in awareness and behavior over time and evaluate the impact of structured educational and digital interventions. Additionally, qualitative studies may help uncover the cultural and psychosocial factors influencing asthma management in this region.

## Conclusions

While general awareness of asthma in Al-Qunfudhah is encouraging, critical gaps persist in symptom recognition, medication use, comorbidity understanding, and healthcare utilization. These findings highlight the urgent need for multifaceted public health interventions that promote not only awareness but also behavioral change and self-management. Targeted educational strategies, integrated care models, and the use of telemedicine could collectively enhance asthma outcomes and reduce the disease burden in semi-urban and rural Saudi communities.
